# Silver ferrite: a superior oxidizer for thermite-driven biocidal nanoenergetic materials†

**DOI:** 10.1039/c8ra08997c

**Published:** 2019-01-14

**Authors:** Tao Wu, Michael R. Zachariah

**Affiliations:** Department of Chemistry and Biochemistry, Department of Chemical and Biomolecular Engineering, University of Maryland College Park MD 20742 USA mrz@umd.edu

## Abstract

Silver-containing oxidizers are of interest as biocidal components in energetic application such as thermites due to their biocidal agent delivery. In this study, AgFeO_2_, was evaluated as an oxidizer in aluminum-based thermite system. This novel oxidizer AgFeO_2_ particles were prepared *via* a wet-chemistry method and its structure, morphologies and thermal behavior were investigated using X-ray diffraction, scanning electron microscopy, thermogravimetric analysis and differential scanning calorimetry, and time-resolved temperature-jump time-of-flight mass spectrometry. The results indicate the decomposition pathways of AgFeO_2_ vary with heating rates from a two-step at low heating rate to a single step at high heating rate. Ignition of Al/AgFeO_2_ occurs at a temperature just above the oxygen release temperature that is very similar to Al/Fe_2_O_3_ and Al/CuO. However, with a pressurization rate three times of Al/CuO, Al/AgFeO_2_ yields a comparable result as to Al/hollow-CuO or Al/KClO_4_/CuO, with a simpler preparation method. The post combustion products demonstrated that the Al/AgFeO_2_ thermite reaction produces a fine dispersion of elemental nanosized silver particles which coats the larger alumina particles and is thus bioavailable.

## Abstract 2

Al/AgFeO_2_ thermite offers very high pressurization rate and delivers bioavailable Ag particles.

## Graphical abstract

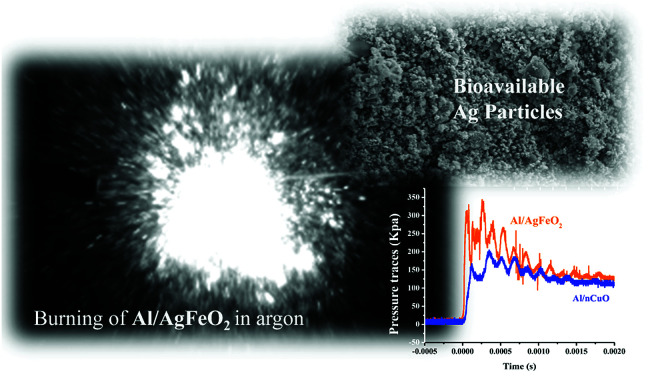

## Introduction

High efficiency neutralization of biological warfare agents has become of increased importance due to the enhanced threat of bioterrorism.^1–4^ Preliminary laboratory studies have suggested that an ideal neutralization process should contain not only a high thermal event but also a long-lasting biocidal agent release.^5–11^ The main problem with conventional energetic materials is low neutralization efficiency since a thermal neutralization mechanism is nominally dominant.^5^ Therefore, it has been proposed that simultaneously delivering a rapid thermal pulse with a remnant biocidal agent would prolong the exposure time and improve the inactivation process.^12^

Reactive materials are a class of energetic materials containing separated fuels and oxidizers and featuring an extremely high exothermicity, intensive light emission and shock generation by the self-sustained reaction.^13–15^ When metal and metal oxide are employed as the fuel and oxidizer, respectively, the intense exothermic metal/metal oxide reaction is commonly referred to a thermite reaction. This reaction is self-propagating once the ignition starts and the kinetics of the reaction are known to be accelerated when the fuel and oxidizer are at the nanoscale, resulting from the increased interfacial contact and reduced characteristic diffusion length scale. The most widely adopted metal is aluminum due to its abundance and competitive reactivity when compared with other metals.^16^ Various oxidizers, such as metal oxides,^17–26^ iodine oxides,^27–32^ sulfates,^33,34^ iodates,^12,35,36^ potassium salts,^37,38^ and *etc.*, have been employed in formulation of thermites. Among all, halogens-containing and silver-containing oxidizers have drawn attention because of their excellent biocidal properties.^39–43^ For halogens, iodine stands out owing to its strong neutralization effect and different iodine-containing oxy-compounds, such as metal iodates and iodine oxides,^5,12,18–25,27–33,35–37,44^ have been studied in thermite systems.

As to silver, it has been pointed out that silver exhibits biocidal properties in the forms of metallic Ag particles and silver ions in a humid environment.^45^ Since most of the products of thermite combustion tend to be primarily in the condensed phase,^2^ metallic silver particles are the focus of this work. As indicated by Morones *et al.*^46^ and Smetana *et al.*,^47^ small particle sizes are necessary for silver particles to perform well in biocidal activities. To deliver not only a high thermal event but also a large amount of small silver particles as the active biocidal sites is the goal of this work.

When it comes to silver-containing oxidizers, Ag_2_O was the obvious choice to be considered as an oxidizer in an aluminum-based thermite system. In 2010, Clark *et al.*^48^ investigated the combustion performance and biocidal abilities of both Al/I_2_O_5_ and Al/Ag_2_O thermites using a homemade biocidal reaction chamber. They concluded Al/I_2_O_5_ thermite exhibited significant spore neutralization owing to the generation of lot of iodine gas. In 2011, Russell *et al.*^49^ further studied the flame propagation behaviors of Al/I_2_O_5_ and Al/Ag_2_O thermites using mechanical impact and thermal initiation. The results show that Al/Ag_2_O features a lower average flame propagation by about 2.5 times in thermal ignition tests but produce much more energy than Al/I_2_O_5_ in impact-driven ignition tests. They also argued that the energy release of the thermite reactions is significantly enhanced by reducing the sizes of the oxidizers particles. In the same year, Sullivan *et al.*^5^ investigated the performance of AgIO_3_ as an oxidizer in aluminum-based thermite because it decomposed to O_2_, O and I gases when heated at an ultra-high heating rate. Silver was not observed in the mass spectra probably because the temperature was not high enough to reach the adiabatic flame temperature and thus could not vaporize silver. However, Al/AgIO_3_ considerably outperformed Al/CuO in pressurization rate due to a large number of gaseous products released from AgIO_3_; however, its high combustion performance was mitigated by the fact that the reaction products were found to form AgI instead of elemental silver and iodine, thus obstructing its usage in biocidal applications.

Sullivan *et al.*^2^ subsequently synthesized nano-Ag_2_O particles and investigated its reactivity as an oxidizer in biocidal energetic systems since it produces high yields of antimicrobial silver as one of the combustion products. They found that Ag_2_O alone performs poorly in terms of pressurization rate and burn time, but its performance is significantly improved when combined with one more reactive oxidizer, such as AgIO_3_ or CuO. The morphology of the final products was also studied and indicated that abundant active sites of silver particles were sacrificed since some silver particles were trapped within the interior of other products, which might to some extent affect its biocidal activity. Inspired by the fact that CuO addition improves the performance of Al/Ag_2_O significantly, we embed the extra oxidizer into Ag_2_O molecularly in this work. Since we have some experience on ferrite-type oxides (AFeO_4_) and delafossite-type oxides (ABO_2_) previously, AgFeO_2_ as a molecularly mixed oxidizer of Ag_2_O and Fe_2_O_3_ with a delafossite structure was a good choice. In addition, Fe_2_O_3_ has been proven to be a poor oxidizer in Al-based thermite system,^37^ it could be of significant interest if AgFeO_2_ which comprises two poor oxides (Ag_2_O and Fe_2_O_3_) became a strong oxidizer.

A wet-chemistry method was adopted here for the preparation of AgFeO_2_ and its thermal behavior were investigated using a low heating rate thermogravimetric analysis and differential scanning calorimetry in an argon environment. Time-resolved temperature-jump time-of-flight mass spectrometry (T-Jump/TOFMS) was also employed to evaluate the decomposition behaviors of bare AgFeO_2_ or Al/AgFeO_2_ thermites under rapid heating rates, enabling us to probe the reaction process on a time scale close to that of a combustion event. The results indicate that the decomposition pathways of AgFeO_2_ vary in term of heating rates. For a comparison purpose, Al/CuO, Al/Ag_2_O and Al/Ag_2_O/Fe_2_O_3_ were also included in this work. A high-speed camera coupled with T-Jump/TOFMS simultaneously captured optical emission from the ignition/reaction of the thermites allowing us to obtain the ignition time, and corresponding ignition temperature. In addition, constant volume combustion cell tests were performed on aluminum-based thermites. The post combustion products were characterized by X-ray diffraction, scanning electron microscopy, transmission electron microscopy and energy-disperse X-ray spectroscopy. The results demonstrated that the Al/AgFeO_2_ thermite reaction produce an enormous amount of nanosized silver particles and feature the best combustion performance in this work.

## Materials and characterizations

### Materials

The aluminum nanopowders (Al) (Alex, ∼80 nm) was purchased from Novacentrix. The active Al was 81% by mass, determined by TGA. All metal oxide nanopowders (<50 nm) purchased from Sigma-Aldrich was directly used as received. All the other chemicals were of analytical grade and used as purchased without further treatment.

### Preparation of AgFeO_2_

AgFeO_2_ powders were prepared *via* a co-precipitation method. For this, 2.66 mmol of Fe(NO_3_)_3_·9H_2_O and 2.66 mmol of AgNO_3_ were dissolved in 20 mL of water and stirred for 30 minutes. The solution was then heated to 80 °C and stirred for one hour. Then, 1.5 M of NaOH solution was added dropwise into the solution until its pH reaches 13, followed by another 6 hours of stirring on a hot plate (80 °C). The prepared AgFeO_2_ powders could be easily isolated from the solution by vacuum filtration and were purified by successively washing with copious amount of distilled water and absolute ethanol. Finally, the product was dried in an oven at 70 °C.

### Preparation of Ag_2_O

Ag_2_O powders were prepared by adding 0.025 M NaOH solution dropwise into 80 mL of AgNO_3_ solution (0.005 M) with stirring until the solution become a grey–yellow colloidal suspension. The suspension was kept at 60 °C for another 2 hours to ensure complete reaction. The prepared Ag_2_O powders were collected by centrifugation and washed with distilled water and then absolute ethanol three times. The solid pure Ag_2_O was obtained after being dried in an oven at 70 °C for 10 hours.

### Preparation of thermites

Aluminum nanopowders was stoichiometric mixed with AgFeO_2_, Ag_2_O, CuO and Fe_2_O_3_ based on the following equations, respectively, in dry hexane followed by 30 minutes sonication (Table 1). After room temperature evaporation of the solvent the solid thermite powders were collected.

**Table 1:** Calculated heat of reaction of thermite reactions^a^

Thermite reaction	Heat of reaction (cal g^−1^)
2Al + 3Ag_2_O → 6Ag (l, g) + Al_2_O_3_ (l)	504.8
2Al + 3CuO → 3Cu (l, g) + Al_2_O_3_ (l)	974.1
2Al + Fe_2_O_3_ → 2Fe (l, g) + Al_2_O_3_ (l)	945.4
4Al + 3AgFeO_2_ → 3Ag + 3Fe + 2Al_2_O_3_ (l)	

a The thermodynamic data of AgFeO_2_ is unknown. Data is taken from Fischer and Grubelich^50^ without taking account of the oxide shell on aluminum.

### T-Jump/TOFMS measurement and high-speed imaging

The decomposition of oxides particles was investigated using a custom T-Jump/TOFMS^5^ Typically, a ∼1 cm long platinum wire (76 μm in width) with a thin coating of oxidizer or thermite sample was rapidly joule-heated to about 1200 °C by a 3 ms pulse at a heating rate of ∼10^5^ °C s^−1^. The current and voltage signals were recorded, and the temporal temperature on the wire was measured according to the Callendar–Van Dusen equation. MS spectra were measured every 0.1 ms. The detailed experimental set-up is given in our previous papers.^5,33^

To identify the point of ignition a high-speed camera (Vision Research Phantom v12.0) was employed to record the combustion on the wire during heating. Ignition temperatures of thermite reactions in vacuum were measured from the correlation of optical emission from high speed imaging and temporal temperature of the wire and were further analyzed in combination with the temporal mass spectra. Each experiment was repeated 3 times.

### X-ray diffraction (XRD) measurement and Rietveld refinement

The as-prepared samples were characterized by powder X-ray diffraction. Diffraction pattern was measured using Cu Kα radiation in Bragg–Brentano geometry on Bruker D8 Advance powder diffractometer equipped with incident beam Soller slits, Ni β-filter and LynxEye position sensitive detector. Data were collected from 10° to 90° 2*θ* with a step size of 0.01578° and counting time of 1 s per step (total exposure time of 180 s per step).

### Thermogravimetric analysis/differential scanning calorimetry (TGA/DSC) measurement

Thermogravimetric analysis and differential scanning calorimetry (TGA/DSC) was performed using a TA Instruments SDT Q600. The analysis was performed under a 100 mL min^−1^ argon flow with ∼1.0 mg samples placed into an alumina pan and heated from room temperature up to 1000 °C at a rate of 10 °C min^−1^ in argon atmosphere.

### Morphologies and structures characterizations transmission electronic microscopy (TEM)

Transmission electronic microscopy (TEM, JEOL JEM 2100 FEG) and scanning electronic microscopy (SEM, Hitachi Su-70) were used to investigate morphologies and structures of thermites. Elemental distribution in the thermites was analyzed by Energy-disperse X-rat spectroscopy (EDS) on both SEM and TEM.

### Combustion test

Combustion properties of themites were evaluated in a constant-volume combustion cell, with simultaneous pressure and optical emission measurements. In this study, 25 mg of thermite powders was loaded inside the cell (constant volume, ∼13 cm^3^) and ignited by a resistively heated nichrome wire. The temporal pressure and optical emission from the thermite reaction were measured using a piezoelectric pressure sensor and a photodetector, respectively. More detailed information on the combustion cell test can be found in our previous publications.^21,25,30^ Each experiment was repeated at least 3 times.

## Results and discussions

### Synthesis and characterization of AgFeO_2_

**Fig. 1:**
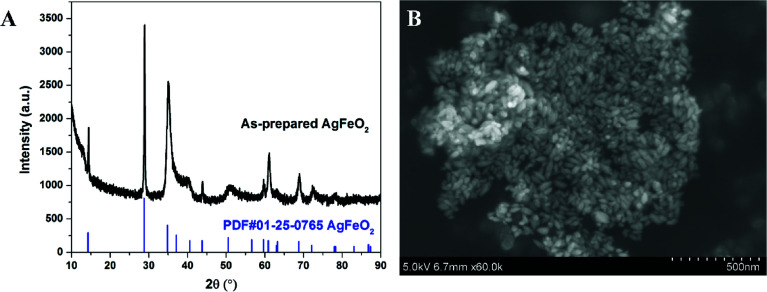
XRD spectrum (A) and SEM image (B) of AgFeO_2_ prepared *via* co-precipitation.

XRD of as-prepared materials shown in Fig. 1A indicates that silver ferrite was successfully prepared. The particles have an oval shape with particle size of ∼40 nm based on the SEM image shown in Fig. 1B, and suggests it might be a very good oxidizer in a thermite system due to its small size.^33^

**Fig. 2:**
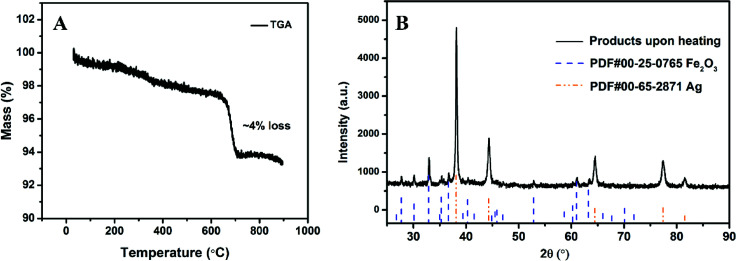
(A) TGA of AgFeO_2_ in Ar at a heating rate of 10 °C min^−1^; (B) AgFeO_2_ decomposition product after heating to 1000 °C.

The thermal stability of AgFeO_2_ was studied with a TGA in Ar at a heating rate of 10 °C min^−1^. The result shown in Fig. 2 indicates a ∼4% weigh loss at around 650 °C, corresponding to O_2_ gas release based on eqn (1). To determine the composition of the remaining material, XRD shown in Fig. 2B indicates the formation of both Fe_2_O_3_ and Ag.

1 2AgFeO_2_ → 2Ag + Fe_2_O_3_ + 1/2O_2_

**Fig. 3:**
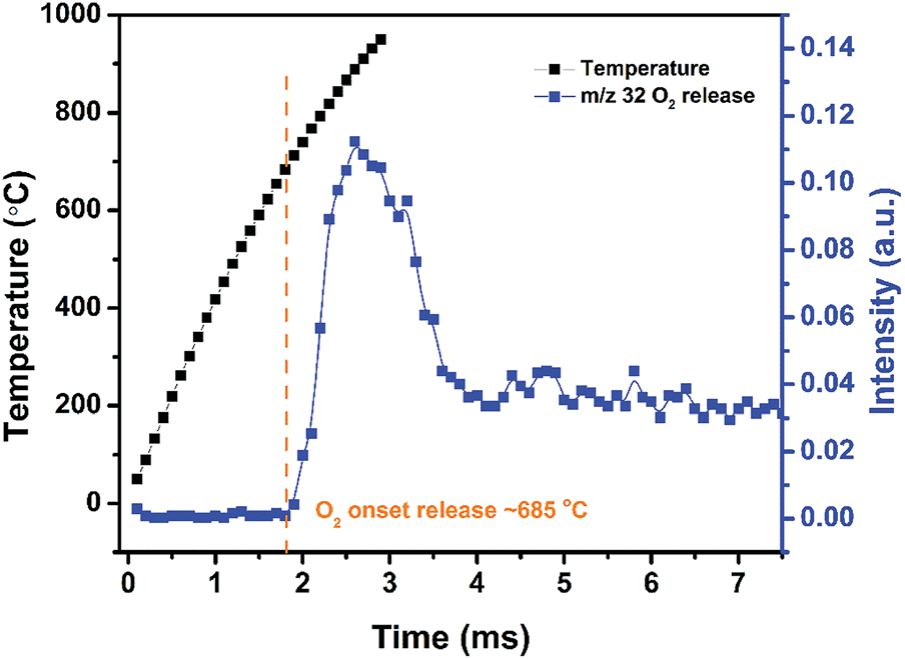
Temperature and O_2_ temporal profile of AgFeO_2_.

High heating rate decomposition of AgFeO_2_ was investigated using TOFMS/T-Jump (3 ms, heating rate ∼ 5 × 10^5^ °C s^−1^). Fig. 3 shows the temporal evolution of O_2_ from AgFeO_2_ during rapid heating. Oxygen was first detected at around 685 °C which is slightly higher than the onset decomposition temperature under low heating rate TGA. Only one O_2_ signal stage is observed indicating the decomposition of AgFeO_2_ is a one-step event at high heating rates conditions, as compared to its multistage decomposition behavior at low heating rates as discussed previously.

Time-resolved T-Jump mass spectra from rapid heating of AgFeO_2_ at 1.6–2.2 ms is shown in Fig. S1† to further explore its decomposition process. Apparently, the onset decomposition of AgFeO_2_ started at around 1.8 ms (685 °C) with the appearance of a small O_2_ peak. For mass spectra taken at prior times, H_2_O^+^, OH^+^, CO^+^/N_2_^+^ peaks are attributed to the background.^18^ Above the decomposition temperature, no new peak except for O_2_ peak is found again suggests a one-step decomposition of AgFeO_2_ at high heating rate. CO_2_^+^ was also observed which we attribute to decomposition products of the precursor salt residue on the AgFeO_2_ surface.^17^

### Ignition of Al/AgFeO_2_ nanothermite

**Fig. 4:**
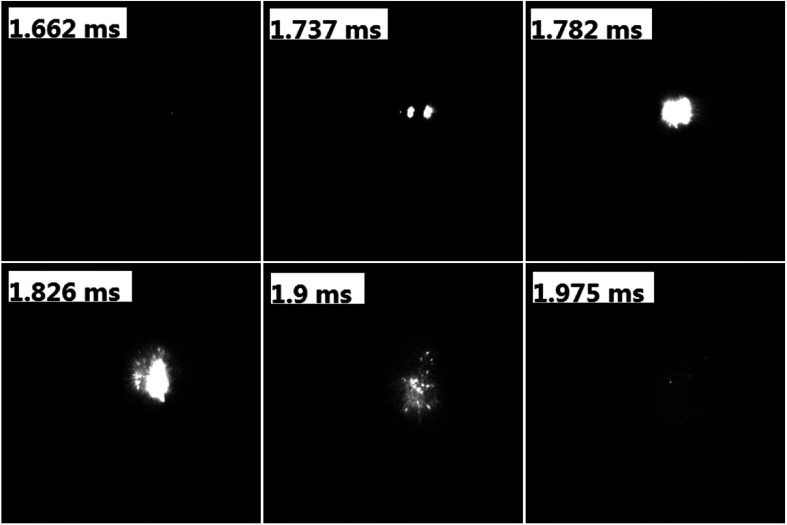
Sequential snapshots of Al/AgFeO_2_ burning on a high-rate heating Pt wire in T-Jump chamber (in vacuum). The labels in each image are the time elapse after triggering.

To evaluate the performance of AgFeO_2_ as an oxidizer in Al-based thermite, physical mixtures of nanosized aluminum and AgFeO_2_ were made, following 30 min sonication of the mixture in dry hexane. Sequential snapshot of Al/AgFeO_2_ ignition during high-rate heating under vacuum were captured using a high-speed camera and is shown in Fig. 4. Optical emission from Al/AgFeO_2_ reaction was first observed at 1.662 ms with a corresponding wire temperature of ∼740 °C as a sign of ignition. Multiple ignition points are observed in the prior time and then merge into a large bright flame. One should also notice that, upon ignition, thousands of bright dots were rapidly ejected out from the reactants coated on the Pt wire (even more bright dots appeared when ignited in an argon environment as shown in Fig. S2†). The burn time of the thermite in T-Jump chamber could be roughly obtained based on the visual flame and is about 0.3 ms, which is almost the same as the value obtained from the combustion cell test discussed below.

Ag_2_O was included as an oxidizer in Al-based thermites as a control. As the most common used oxidizer, CuO was also included as a standard reference. Since AgFeO_2_ (Ag_2_Fe_2_O_4_) can be molecularly written as Ag_2_O and Fe_2_O_3_, a binary mixture Ag_2_O–Fe_2_O_3_ with 1/1 molar ratio is also included for comparison.

**Fig. 5:**
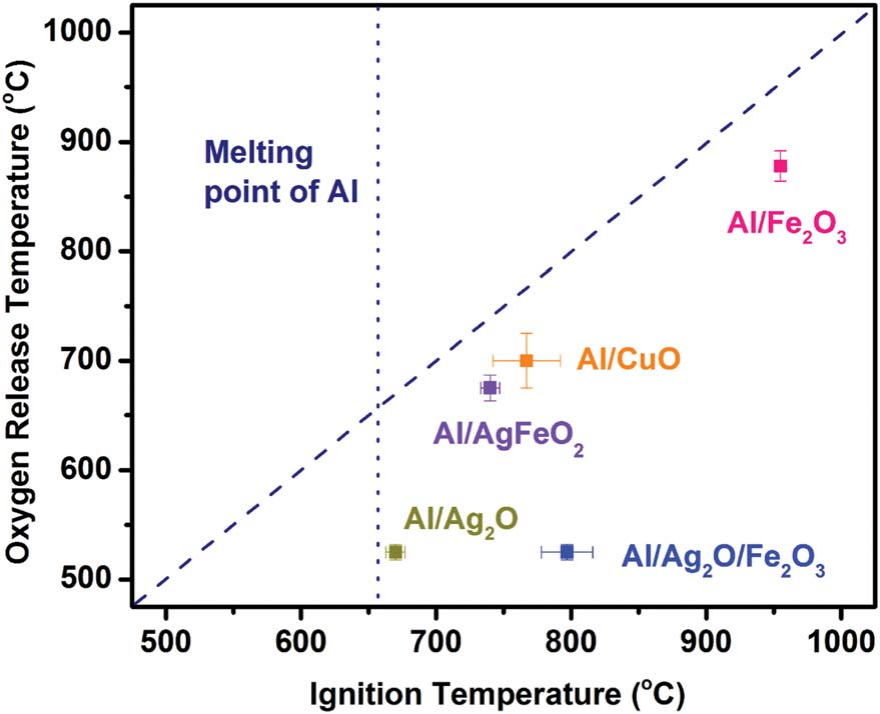
Ignition temperature *vs.* oxygen release temperature from the corresponding oxidizer. The diagonal dash line presents a perfect correlation.

Fig. 5 shows the relationship between O_2_ release temperature in neat oxides and the ignition temperature of corresponding thermites under vacuum. There is a good correlation between the oxygen release from the bare oxidizer and ignition of Al/CuO, Al/Fe_2_O_3_ and Al/AgFeO_2_. Al/Fe_2_O_3_ has a very high ignition temperature due to the poor performance of Fe_2_O_3_ as an oxidizer.^24^ On the other hand, Al/Ag_2_O thermite ignited at around ∼660 °C, essentially the melting temperature of aluminum. Considering Ag_2_O releases oxygen at around 520 °C, it is reasonable to conclude that the ignition of Al/Ag_2_O thermite is limited to the melting phase of aluminum like most Al/metal oxides systems.^19^ In fact, the ignition temperatures of the other four samples are all higher than the melting point of aluminum indicates that gaseous oxygen released from the decomposition of oxidizers, was insufficient to ignite aluminum when it is still in the solid phase.

Al/Ag_2_O/Fe_2_O_3_ ternary thermite ignited at around 800 °C much higher than its oxygen release temperature (520 °C). Considering Al/Ag_2_O and Al/Fe_2_O_3_ ignited at around 660 and 940 °C, respectively, an ignition temperature of about 800 °C is reasonable for the ternary system Al/Ag_2_O/Fe_2_O_3_. The Fe_2_O_3_ addition is more likely hindering/weakening the ignition/reaction of the ternary system due to its weak reactivity in Al-based thermites.^37^

As to Al/AgFeO_2_, it ignited at a temperature close to the oxygen release temperature of the corresponding oxidizer and higher than the melting temperature of aluminum following an ignition mechanism similar to Al/Fe_2_O_3_ and Al/CuO.^18,19^ Moreover, the fact that the ignition temperature of Al/Fe_2_O_3_ is much higher than that of Al/AgFeO_2_ implies the oxygen involved with Al/AgFeO_2_ ignition come from AgFeO_2_ rather than the first-stage decomposed product Fe_2_O_3_ as indicated in TGA. Since only one oxygen release stage appeared at the mass spectra and the violent reaction of Al/AgFeO_2_ at 1.7 ms (∼780 °C) implies decomposition of AgFeO_2_ is a one-step process during Al/AgFeO_2_ thermite reaction at high heating rate.

### Combustion performance of Al/AgFeO_2_ nanothermite

**Fig. 6:**
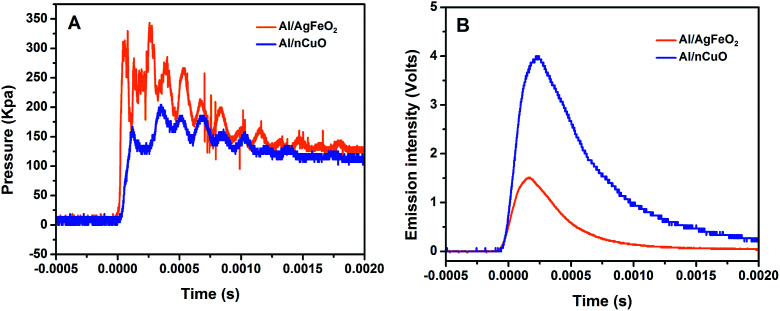
Temporal pressure and optical behavior of Al/AgFeO_2_ and Al/*n*CuO.

The combustion performance of Al/AgFeO_2_ at room atmosphere was studied using a constant volume combustion cell, and the results are summarized in Table 2. The pressurization rate and peak pressure in Al/AgFeO_2_ reaction are 6200 kPa ms^−1^ and 325 kPa, respectively, which are considerably higher than those of nano-thermite reaction of Al/CuO. A direct comparison between the temporal pressure traces of Al/AgFeO_2_ and Al/CuO was shown in Fig. 6A in which the first peak was used to determine the corresponding pressurization rate. Clearly, Al/AgFeO_2_ reaction reaches its first peak with a faster rate and higher peak value compared with Al/CuO. We have claimed in our previous works that gaseous species release from the decomposition of the oxidizer is the main cause for the pressurization, which can occur much earlier than ignition/combustion.^37,51^ The fact that AgFeO_2_ (∼16%) has a lower oxygen weight ratio than CuO (∼20%) suggests the O_2_ release rate of AgFeO_2_ must be higher than that of CuO in order to output such high pressure. In addition, the optical signals peak much later than the corresponding pressure peaks for both Al/CuO (∼0.5 ms) and Al/AgFeO_2_ (0.9 ms) implying a similar mechanism.^51,52^ Therefore, similar to Al/CuO,^37^ the ignition/reaction mechanism of Al/AgFeO_2_ is summarized as follows: AgFeO_2_ releases O_2_ gas (∼690 °C) and the aluminum core becomes mobile (633 °C); and the mixture ignites (∼740 °C, Fig. 5) and the generated heat further promote the decomposition of AgFeO_2_ to pressurize the system.

**Table 2:** Combustion performance of mixed thermites of Al/AgFeO_2_

Thermites (stoichiometric)	Peak pressure (kPa)	Pressurization rate (kPa ms^−1^)	Burn time (ms)	Peak optical emission (volts)
Al/*n*CuO	226	2045	0.46	4.3
Al/Ag_2_O	75	25.7	3.6	0.3
Al/Ag_2_O/Fe_2_O_3_	74	21	5	0.4
Al/AgFeO_2_	**325**	**6200**	0.3	1

Moreover, with a pressurization rate three times of Al/CuO, Al/AgFeO_2_ yields a comparable result as prior work on Al/hollow-CuO^25^ or Al/KClO4/CuO,^37^ which make AgFeO_2_ a powerful replacement owing to its simpler and cheaper preparation method.

Sullivan *et al.*^2^ has previously reported that Al/Ag_2_O suffers a poor combustion performance in terms of both pressurization rate and peak pressure and incorporation of a small fraction of CuO into the Al/Ag_2_O system improved its combustion performances extensively to even close to the reactivity of Al/CuO. An explanation was provided by Sullivan *et al.* that CuO addition can increase the reaction temperature and thus further enhance the performance of Ag_2_O as an oxidizer. Consistent with this reported result, Al/Ag_2_O performs poorly in the combustion cell test due to an early release of O_2_;^2^ however, Fe_2_O_3_ addition does not show much improvement to Al/Ag_2_O system as CuO does. Instead of improving, Fe_2_O_3_ even weaken the combustion performance of Al/Ag_2_O system considering Al/Ag_2_O/Fe_2_O_3_ features the worst pressurization rate and burn time. Thermodynamic equilibrium predictions of Al/CuO, Al/Ag_2_O and Al/Fe_2_O_3_ thermites by Fischer and Grubelich^2,27,50^ are shown in Table 3 where we can see that both CuO and Ag_2_O should perform significantly better than Fe_2_O_3_ in terms of gas production. And this is consistent with our experimental data shown in Table 2. In detail, unlike CuO that can rapidly release O_2_, Fe_2_O_3_ cannot efficiently decompose and upon decomposition most of the oxygen is still fixed as solid FeO,^37^ which leads to its decomposition as the rate-limiting step for Al/Ag_2_O/Fe_2_O_3_ reaction and therefore weakened combustion performance. In fact, Fe_2_O_3_ has been introduced previously as a moderator to weaken the reactivities of Al/KMnO_4_ ^24^ and Al/KClO_4_.^37^

**Table 3:** Thermodynamic equilibrium predictions of Al/CuO, Al/Ag_2_O and Al/Fe_2_O_3_ thermites^a^

Thermite reaction	Adiabatic temperature (°C)	Gas production (mmol g^−1^)	Major gas species
2Al + 3CuO → Al_2_O_3_ (l) + 3Cu (l, g)	2570	5.4	Cu
2Al + 3Ag_2_O → Al_2_O_3_ (l) + 6Ag (l, g)	2163	4.3	Ag
2Al + Fe_2_O_3_ → Al_2_O_3_ (l) + 2Fe (l, g)	2862	1.4	Fe

a Assumptions: constant enthalpy and pressure with phase changes; without taking account of the oxide shell of aluminum. Data is taken from Fisher and Grubelich.^50^

Al/AgFeO_2_ significantly outperforms Al/Ag_2_O/Fe_2_O_3_ by a scale of almost 100 in pressurization rate and about 4 in peak pressure. The fact that Al/AgFeO_2_ and Al/Ag_2_O/Fe_2_O_3_ share the exact same elemental compositions indicates molecularly incorporation of Ag_2_O into Fe_2_O_3_ outperforms the mechanically mixed Ag_2_O/Fe_2_O_3_ when they were employed as oxidizers in aluminum-based thermites.

The burn time of the thermite reactions measured in the combustion cell are shown in Table 2. Clearly, Al/AgFeO_2_ features the shortest burn time which makes it the most violent thermite among the four examined. A direct comparison of the optical emission trace of Al/AgFeO_2_ and Al/CuO was shown in Fig. 6B. The Al/CuO reaction has a four-times higher peak optical emission but 0.1 ms longer burn time compare with those of Al/AgFeO_2_. This result indicates that Al/AgFeO_2_ is a weaker heat generator than Al/CuO; but reacts more rapidly. In general, AgFeO_2_ is the best oxidizer among those four in the aluminum-based thermite system from both pressurization and optical emission perspectives.

### Post-combustion-product characterization

**Fig. 7:**
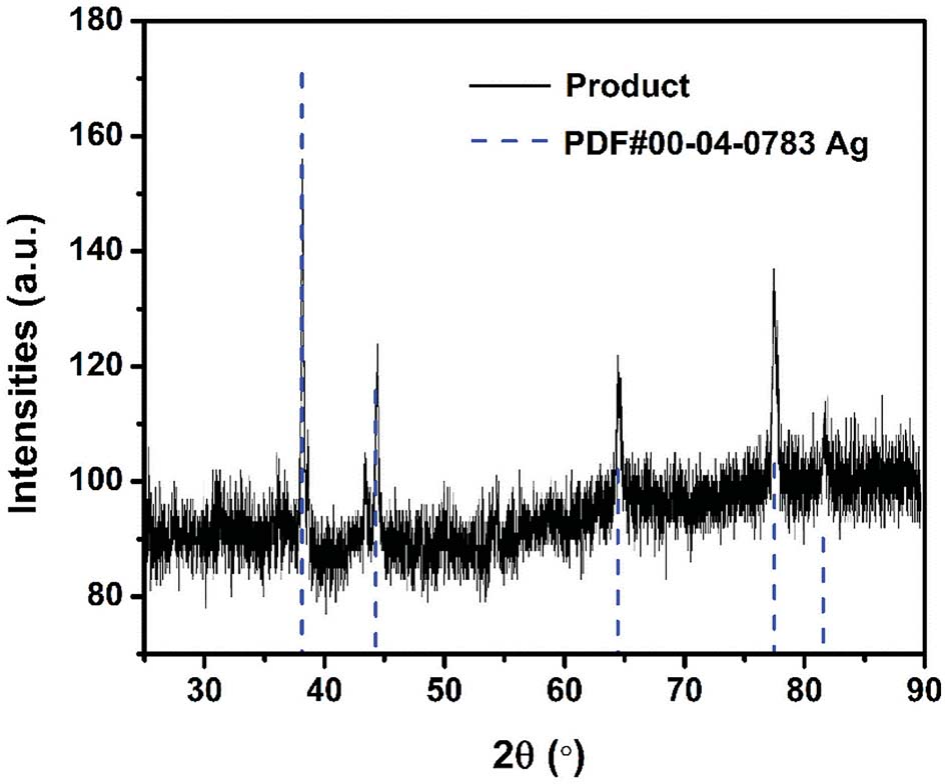
The XRD pattern of Al/AgFeO_2_ reaction product.

It has been pointed out previously that the nature and dispersion of the products from a thermite combustion plays an important role in biocidal applications.^27^ XRD evaluation of crystalline product species is shown in Fig. 7 and show no evidence of the parent starting materials, but five new strong peaks indexed to elemental silver are seen. The fact that no peak corresponding to Al_2_O_3_ or Fe was observed might suggest they are both amorphous.

**Fig. 8:**
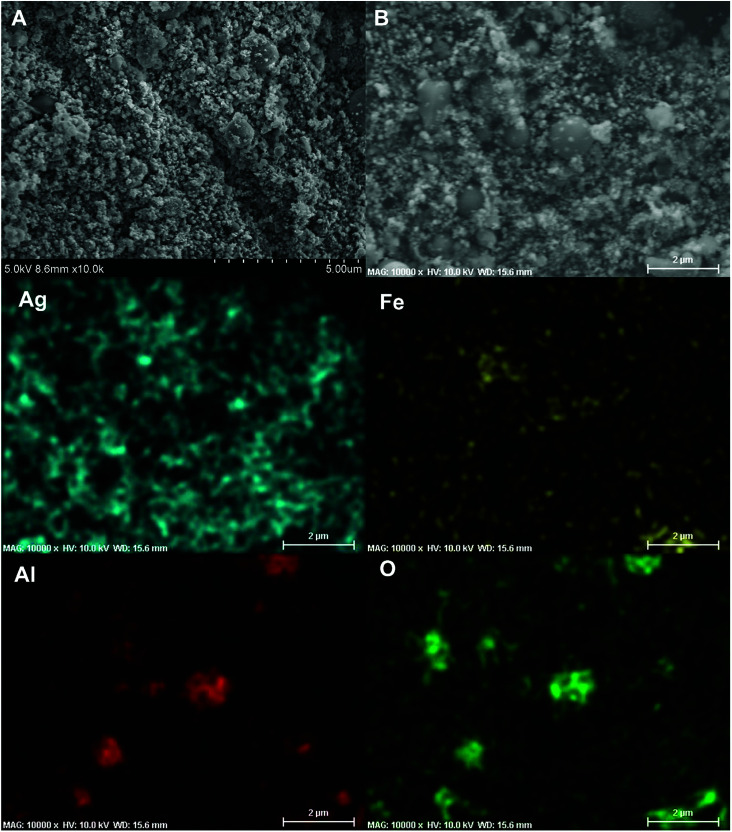
Representative SEM images (A and B) of the Al/AgFeO_2_ reaction product collected inside the combustion cell; 2D elemental mapping (Ag, Fe, Al and O) of (B) using EDS.

For SEM evaluation of the reaction product a 7 mm × 5 mm rectangular double-sided carbon tape was placed inside the combustion cell chamber. Two representative SEM images of the product were shown in Fig. 8A and B, there are mainly two populations of product particle sizes. One has relatively larger dimension and another with a dimension as small as ∼80 nm. 2D elemental mapping using EDS shown in Fig. 8, indicate the larger spherical particles are Al_2_O_3_ while the smaller particles are Ag. This was also confirmed with 1D elemental line-scan coupled with elemental analysis shown in Fig. S3.† Most importantly for the application as a biocide, is that the smaller Ag particles randomly decorate the larger Al_2_O_3_ particles.

Overall, the intensity of iron signal is the weakest among those four elements under EDS mapping implying the random distribution of iron.

**Fig. 9:**
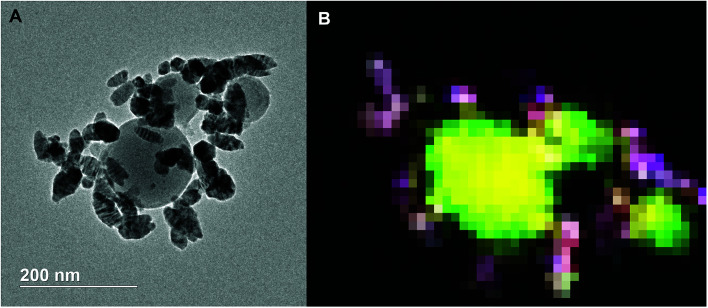
TEM image (A) and 2D elemental mapping (B) of the Al/AgFeO_2_ reaction product. Red represents Ag, blue represents Fe, yellow represents Al and green represents O.

TEM of the reaction product along with an EDS elemental mapping result is shown in Fig. 9. As can be seen, there are two different types of particles similar to the SEM result. One has a spherical shape with a relatively larger particle size and low contrast; another has a spherical/oval shape with smaller size and dark contrast. In the EDS elemental mapping, red represents silver, blue iron, yellow aluminum and green oxygen. Clearly, the three larger spherical particles are Al_2_O_3_. Since the mixing of red and blue gives purple, it is reasonable to conclude that the silver and iron positions are mostly overlapped. It could mean a formation of Ag–Fe alloy; however, the SEM results indicate the iron signal is much weaker and always appears with the presence of both aluminum and oxygen signals. To clarify this speculation, an EDS line-scan analysis was employed, and the result will be discussed below. It should also be noted that some white pixels were found in the purple area, implying the presence of oxygen (green plus red and blue equals to white). Thus, those silver- and iron-containing particles might be partially oxidized that could be a result from handling the product in air.

**Fig. 10:**
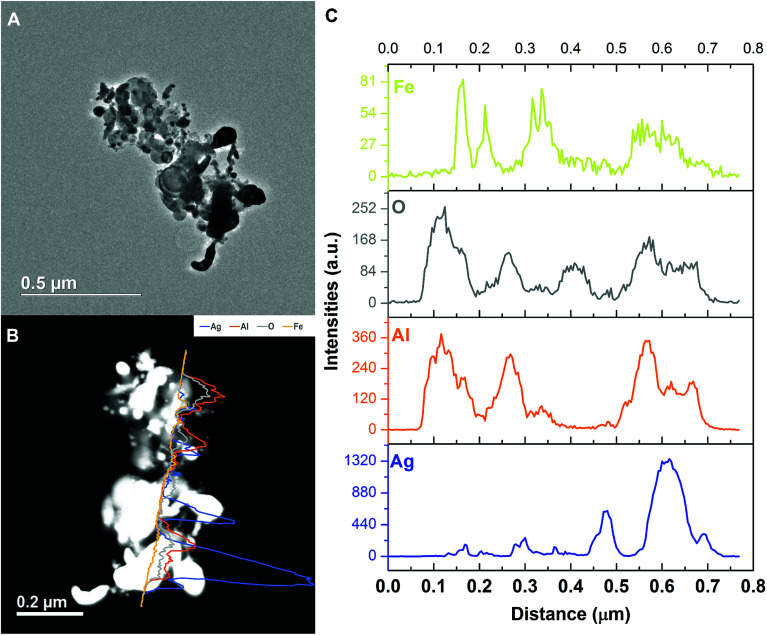
Representative TEM image and the corresponding EDS line-scan data of the Al/AgFeO_2_ reaction product.

As we can see from the EDS line-scan result shown in Fig. 10, excerpt for the small oxygen signal peak at ∼0.4 μm, the aluminum and oxygen signals are almost synchronous in positions indicating Al_2_O_3_. Like the SEM EDS mapping result, iron is the weakest among all four elements. It overlaps with aluminum and silver, respectively, implying the iron is randomly distributed and excluding the hypothesis of Ag–Fe alloy formation.

Comparing these two representative TEM images Fig. 9A and 10A, the morphologies of the silver particles are quite different. The ones in Fig. 9A are nanosized with spherical/oval shapes and are decorated on/near the Al_2_O_3_ surface that suggests silver product had vaporized and re-condensed.^2^ This mechanism is beneficial to biocidal applications due to the wide distribution of small sized silver particles during the violent combustion event. As to the one in Fig. 10A, particles at the bottom right with oddly shapes clearly underwent some sintering. Those results are consistent to a previously reported sintering reaction mechanism of Al-based thermites.^53,54^ However, it seems much less reactants were following the sintering mechanism according to the SEM image shown in Fig. 8 where the majority of silver product has small particle size and distributed randomly.

## Conclusions

In this study, the AgFeO_2_ was prepared *via* a wet-chemistry method to yield phase pure ∼40 nm particles. The decomposition pathways of AgFeO_2_ were found to depend on heating rates: decomposition to Ag, O_2_ and Fe_2_O_3_ at ∼600 °C at low heating rate and direct decomposition ∼685 °C at high heating rates.

The ignition of Al/AgFeO_2_ was found to slightly higher than the oxygen release temperature and thus with a similar mechanism to Al/Fe_2_O_3_ although ignites at a much lower temperature. Fast video imaging indicates a fine smoke dispersing particle.

Combustion cell test showed that Al/AgFeO_2_ outperformed other thermites in maximum pressure, pressurization rate and burn time. Moreover, with a pressurization rate three times and 5% less oxygen content of Al/CuO, Al/AgFeO_2_ yields a comparable result as to Al/hollow-CuO or Al/KClO_4_/CuO. The fact that Al/AgFeO_2_ and Al/Ag_2_O/Fe_2_O_3_ share the exact same elemental compositions but feature the highest and lowest pressurization rate, respectively, indicates molecularly incorporation of Ag_2_O into Fe_2_O_3_ outperforms the mechanically mixed Ag_2_O/Fe_2_O_3_ when they were employed as oxidizers in aluminum-based thermites. Post combustion products indicate the formation of elemental silver nanoparticles (∼<80 nm) decorating larger Al_2_O_3_ and is this bioavailable.

## Conflicts of interest

There are no conflicts to declare.

## Supplementary Material

RA-009-C8RA08997C-s001
