# Supplementary material for: Silver ferrite: a superior oxidizer for thermite-driven biocidal nanoenergetic materials
Source: RSC Adv. 2019 Jan 14;9(4):1831–40. doi: 10.1039/c8ra08997c (PMC9059743; doi:10.1039/c8ra08997c)
Supplement: RA-009-C8RA08997C-s001 [file RA-009-C8RA08997C-s001.pdf]

Electronic Supplementary Material (ESI) for RSC Advances.
This journal is © The Royal Society of Chemistry 2018

Silver Ferrite: a superior oxidizer for thermite-driven biocidal nanoenergetic materials

Tao Wu and Michael R. Zachariah*

* Address all correspondence to this author.
e-mail: mrz@umd.edu

Department of Chemistry and Biochemistry and Department of Chemical and Biomolecular Engineering, University of Maryland, College Park, MD 20742, USA

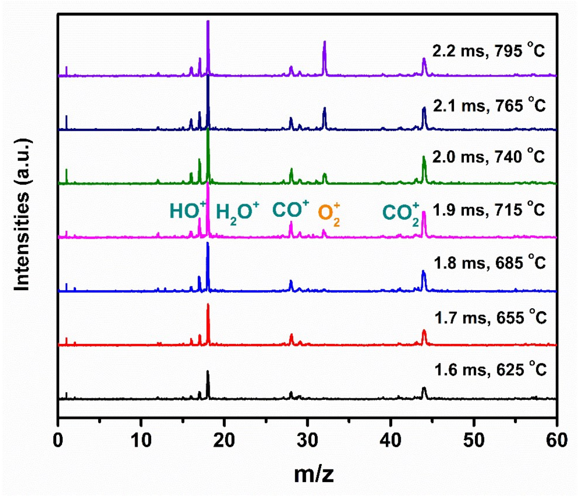

Figure S1. Multiple mass spectra of $AgFeO_2$

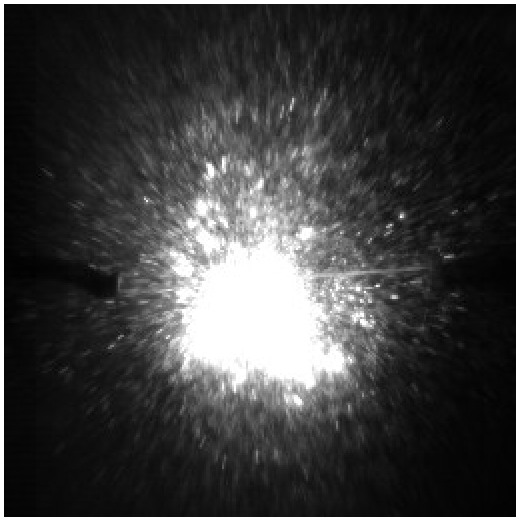

Figure S2. A snapshot of Al/$AgFeO_2$ burning on a high-rate heating Pt wire in T-Jump chamber (in argon).

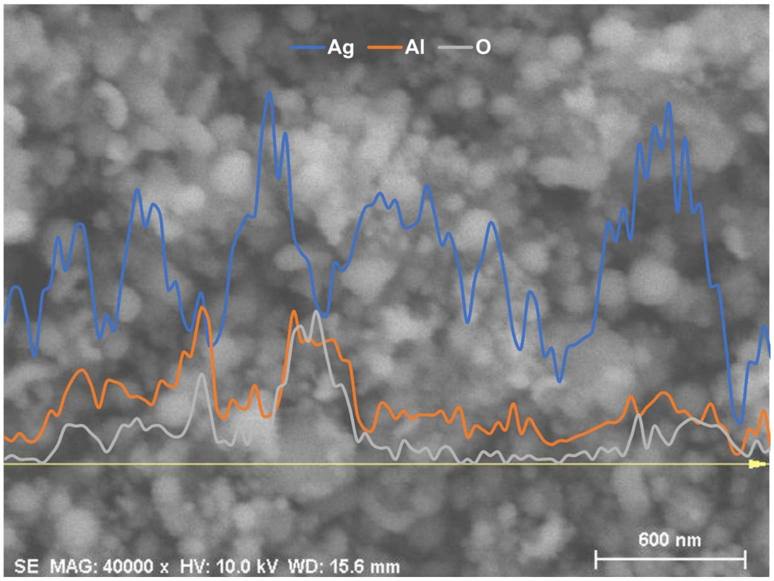

Figure S3. One-dimensional elemental line-scan using EDS of the Al/$AgFeO_2$ reaction product collected inside the combustion cell.
